# New sesquiterpenes from the soft coral *Litophyton arboreum*

**DOI:** 10.1007/s11418-024-01843-w

**Published:** 2024-10-22

**Authors:** Amany Hamouda Mahmoud, Sabry A. H. Zidan, Mamdouh Nabil Samy, Abdallah Alian, Mahmoud A. A. Ibrahim, Mostafa Ahmed Fouad, Mohamed Salah Kamel, Katsuyoshi Matsunami

**Affiliations:** 1 Department of Pharmacognosy, Graduate School of Biomedical and Health Sciences, Hiroshima University, 1-2-3 Kasumi, Minami-ku, Hiroshima, 734-8553 Japan; 2 Department of Pharmacognosy, Faculty of Pharmacy, Minia University, Minia, 61519 Egypt; 3 Department of Pharmacognosy, Faculty of Pharmacy, Al-Azhar University, Assiut-Branch, Assiut, 71524 Egypt; 4 Department of Zoology, Faculty of Science, Al-Azhar University, Assiut-Branch, Assiut, 71524 Egypt; 5 Chemistry Department, Faculty of Science, Minia University, Minia, 61519 Egypt; 6 School of Health Sciences, University of KwaZulu-Natal, Westville, 4000 Durban South Africa

**Keywords:** *Litophyton arboreum*, Nephtheidae, Soft corals, Sesquiterpenes, Cytotoxicity, Anti-leishmania

## Abstract

Two new sesquiterpenes; 8α,11-dihydroxy-β-cyperon (**2**), and 5-*epi*-7α-hydroxy-( +)-oplopanone (**3**), were isolated from the soft coral *Litophyton arboreum,* together with nine known ones, including five sesquiterpenes; 11-hydroxy-8-oxo-β-cyperon (**1**), alismoxide (**4**), 5β,8β-epidioxy-11-hydroxy-6-eudesmene (**5**), chabrolidione B (**6**), 7-oxo-tri-nor-eudesm-5-en-4β-ol (**7**), two sterols; 7β-acetoxy-24-methyl-cholesta-5,24(28)-diene-3β,19-diol (**8**), nebrosteroid M (**9**), and two glycerol derivatives; chimyl alcohol (**10**) and batyl alcohol (**11**). The structures of the isolated compounds were characterized using spectroscopic techniques, predominately HR-ESI–MS, 1D, 2D-NMR, and ECD analyses. Compounds **1**–**11** were evaluated for their cytotoxic activity against three human cancer-cell lines (A549, MCF-7 and HepG2), and anti-leishmanial potential against the causal parasite, *Leishmania major.* Compounds **4**, **8**, and **9** exhibited potent cytotoxic activity against the A549 cell line (IC_50_ = 17.0 ± 2.5, 13.5 ± 2.1, and 16.5 ± 1.3 μg/ml, respectively) as compared with the standard antitumor agent etoposide (IC_50_ 28.4 ± 4.5 μg/ml). In addition, compound **9** exhibited remarkable cytotoxic activity against MCF-7 cell line (IC_50_ = 24.7 ± 2.1 μg/ml: 22.2 ± 4.2 μg/mL for etoposide).

**Graphical abstract:**

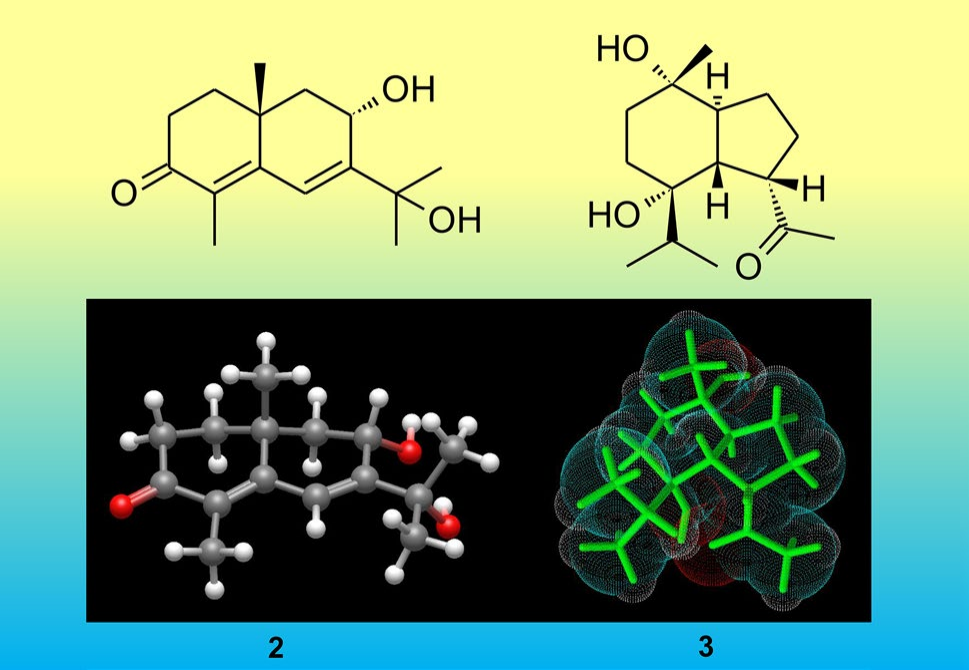

**Supplementary Information:**

The online version contains supplementary material available at 10.1007/s11418-024-01843-w.

## Introduction

Natural products derived from marine sources exhibit a diverse array of biologic activities, which are crucial in the discovery of significant molecules for drug discovery [1]. Soft corals have a distinct class of metabolites with a wide range of bioactivities and impressive structural diversity. Therefore, a chemical study of marine soft corals will yield a large number of chemically diverse compounds with various biologic activities that may be useful in the pharmaceutical field [2]. Nephtheidae, a family of soft corals with twenty species, is a significant source of metabolites with therapeutic potential [3]. The major metabolites that have been identified are terpenes and steroids, which have a variety of biologic properties, including antibacterial, anti-inflammatory, and anticancer properties [4]. The genus *Litophyton* (Syn; *Nephthea*) is an important member of the family Nephtheidae, which is found mostly in the Indo-Pacific area and the Red Sea [5, 6]. The genus *Litophyton* has yielded up to 250 bioactive compounds, mostly sesquiterpenes, diterpenes, and polyhydroxylated steroids [7]. These secondary metabolites have been found to have interesting biologic activity, particularly in cancer treatment, even with modest changes in their structures affecting their potency and selectivity [7]. Few investigations on the chemical and biologic assessments of *L. arboreum* have been conducted [4, 8–10]. Therefore, the phytochemical contents of *Litophyton arboreum* collected in the Red Sea have been investigated, which resulted in the identification of many classes of secondary metabolites, including two previously undescribed compounds.

Human lung cancer is one of the leading causes of death, and Leishmaniasis is designated by WHO as NTDs (Neglected Tropical Diseases) to be tackled promptly. In continuing our research to discover the anticancer and anti-leishmania agents [11–13], the isolated compounds were also evaluated for these activities.

## Results and discussions

### Isolation and structural elucidation

**Fig. 1:**
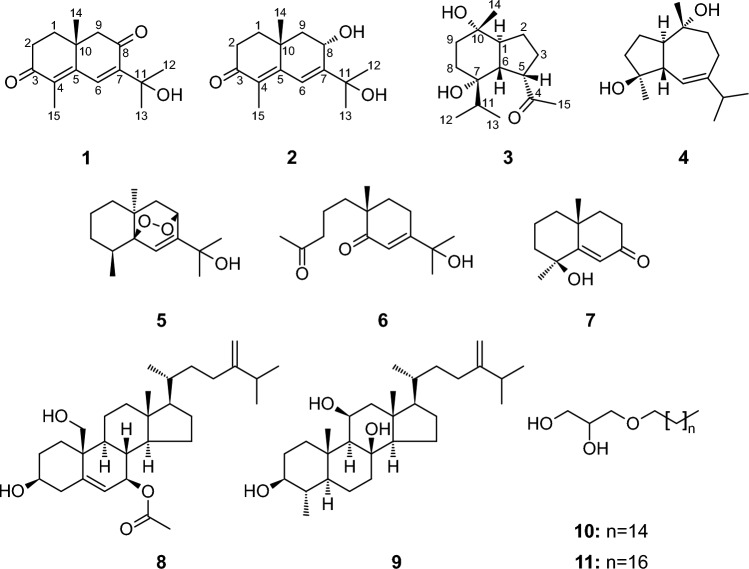
Structures of the isolated compounds **1**–**11** from *L. arboreum*

The methanol extract of soft corals *L. arboreum* collected from the Egyptian Red Sea coast was fractionated by silica gel column chromatography to obtain five fractions eluted with combinations of the organic solvents with increasing polarity. These sub-fractions were further separated and purified using reversed-phase open column chromatography and HPLC (ODS) to yield eleven compounds (**1**–**11**) as shown in Fig. 1, including seven sesquiterpenes; 11-hydroxy-8-oxo-β-cyperon (**1**) [14], 8α,11-dihydroxy-β-cyperon (**2**), 5-*epi*-7α-hydroxy-( +)-oplopanone (**3**), alismoxide (**4**) [10], 5β,8β-epidioxy-11-hydroxy-6-eudesmene (**5**) [15], chabrolidione B (**6**) [16], 7-oxo-tri-nor-eudesm-5-en-4β-ol (**7**) [17], two sterols; 7β-acetoxy-24-methyl-cholesta-5,24(28)-diene-3β,19-diol (**8**) [10], nebrosteroid M (**9**) [18], two glycerol derivatives; chimyl alcohol (**10**) [10]; batyl alcohol (**11**) [19]. Their structures were identified by intensive spectroscopic analyses, in addition to comparing their physical and chemical properties with those reported.

**Fig. 2:**
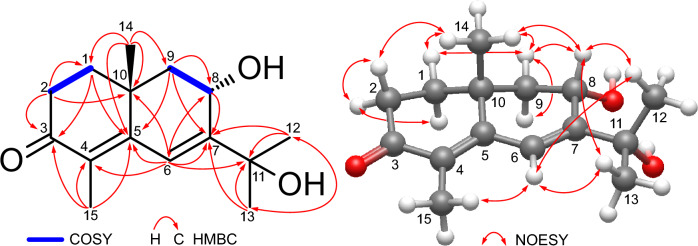
COSY, HMBC and NOESY correlations of **2**

**Fig. 3:**
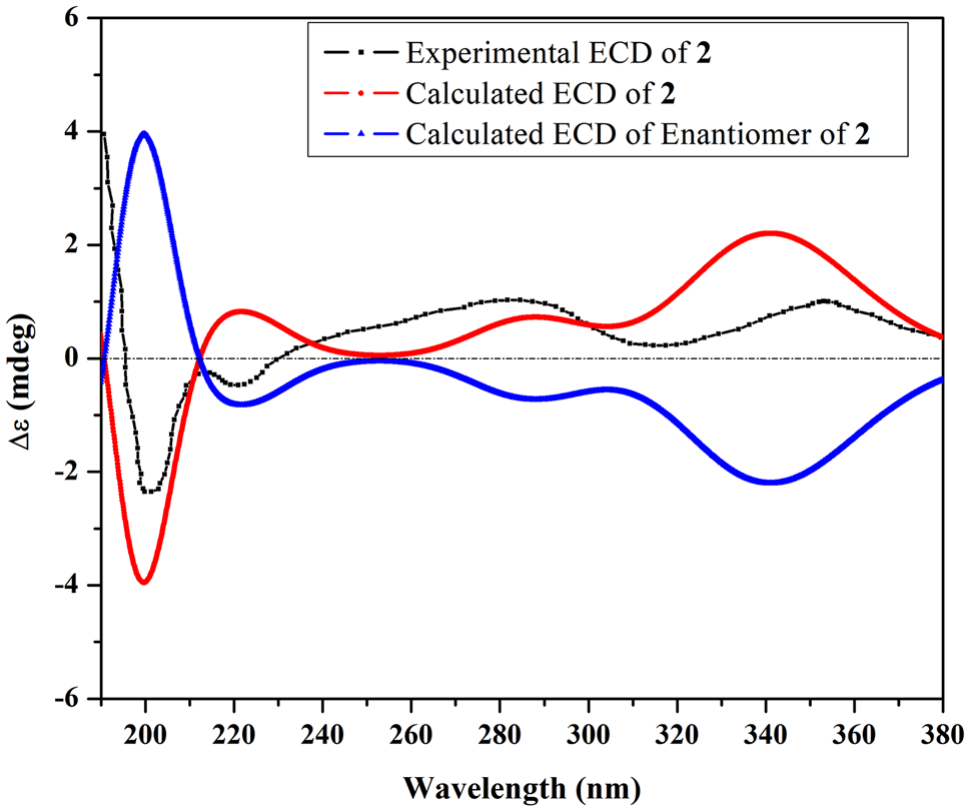
Comparison of the experimental and TDDFT-simulated ECD spectra of **2**

Compound **2**, [α]_D_^23^ + 96.15 (*c* 2.6, MeOH), exhibited a molecular formula of C_15_H_22_O_3_ determined by positive ion-mode HR-ESI–MS that showed a sodiated molecular ion peak at *m/z*: 273.1461 [M + Na]^+^ (Calcd for C_15_H_22_O_3_Na^+^: 273.1461). The IR spectrum of **2 **displayed an absorption band at 3393 cm^−1^ for hydroxyl groups. The UV spectrum showed an absorption band at 296 nm for a conjugated system. The NMR data of compound **2** were closely related to compound **1** [14], except for the chemical shift of C-8 that was highly upfield-shifted from 198.6 in compound **1** to 66.8 in compound **2**, suggesting the presence of a hydroxyl group at C-8. The ^1^H-NMR spectrum of compound **2** (Table 1) showed one doublet olefinic proton signal at *δ*_H_ 6.95 (d, *J* = 1.7 Hz) due to long-range coupling with H-8 assignable for H-6. The four singlet methyl signals at *δ*_H_ 1.11, 1.85, 1.88 and 2.02 were assignable for H_3_-14, 13, 12, and 15, respectively. The ^13^C-NMR and DEPT spectra (Table 1) showed the characteristic carbon signals at *δ*_C_ 198.5 (C-3), 158.1 (C-7), 155.4 (C-5), 128.8 (C-4) and 120.3(C-6), confirming that compound **2** was an eudesmane-type sesquiterpenoid with 4,6-dien-3-one moiety as **1** [14]. In addition, four methyls at *δ*_C_ 32.0, 30.7, 22.4, and 11.2 were assignable for C-12, C-13, C-14, and C-15, respectively. Three methylenes at *δ*_C_ 36.6 (C-1), 34.2 (C-2) and 48.8 (C-9), a quaternary carbon at *δ*_C_ 35.9 (C-10), a hydroxylated methine carbon at *δ*_C_ 66.8 (C-8), and a hydroxylated quaternary carbon at *δ*_C_ 74.4 (C-11) were also assigned by comparing with the chemical shift values of **1**. The HSQC and ^1^H-^1^H COSY analyses were conducted to determine the ^1^*J*_CH_, ^2^*J*_HH_ and ^3^*J*_HH_ connectivities. The HMBC correlations from H-8 with C-6 and 7, from H-6 with C-4, 5, 7, 8, 10, and 11, from H-12/13 with C-7 and 11, from H-14 with C-1, 5, 9 and 10, and from H-15 with C-3, 4 and 5 confirmed the planar structure of **2** (Fig. 2). The axial-axial coupling of H-9α (*δ*_H_ 1.91, dd, *J* = 12.4, 10.6 Hz) indicated the β-axial configuration of H-8. The NOE correlation between H_3_-14 and H-8 confirmed that they have the same orientations. The absolute configuration of C-8 and C-10 is determined to be 8*S* and 10*R* by ECD calculation, as shown in Fig. 3. Consequently, from the above results, compound **2** was revealed to be a previously undescribed compound, namely 8α,11-dihydroxy-β-cyperon.

**Table 1:** ^1^H and ^13^C NMR spectral data of compounds **1**–**3** (600 and 150 MHz, respectively)

No	1^a^^,*^		2^a^		3^b^	
	*δ*_*C*_	*δ*_*H*_ (J inHz)	*δ*_*C*_	*δ*_*H*_ (*J* inHz)	*δ*_*C*_	*δ*_*H*_ (*J* inHz)
1	36.1	1.62 (ddd, 13.2, 5.2, 2.0) 1.85 (m)	36.6	1.62 (ddd, 12.8, 5.4, 2.0) 1.77 (m)	51.8	2.08 (td, 12.5, 5.4)
2	34.3	2.51 (ddd, 17.6, 4.7, 2.0) 2.68 (ddd, 17.6, 15.2, 5.2)	34.2	2.47 (ddd, 17.8, 5.0, 2.0) 2.68 (ddd, 17.8, 15.0, 5.4)	26.6	1.32 (m) 1.80 (quint-like, 6.1)
3	198.3	–	198.5	–	30.4	1.53 (m) 1.90 (qd-like, 11.7, 7.4)
4	134.5	–	128.8	–	215.1	–
5	152.3	–	155.4	–	50.7	3.07 (ddd, 11.7, 8.8, 4.2)
6	149.7	8.16 (s)	120.3	6.95 (d, 1.7)	50.4	2.15 (dd, 12.5, 8.8)
7	135.5	–	158.1	–	75.7	–
8	198.6	–	66.8	5.13 (m)	29.0	1.39 (m)
9	54.7	2.42 (d, 15.0) 2.58 (d, 15.0)	48.8	1.91 (dd, 12.4, 10.6) 2.16 (dd, 12.4, 5.5)	38.1	1.54 (m)
10	38.1	–	35.9	–	73.7	1.51 (m) 1.74 (td, 12.7, 4.3)
11	71.6	–	74.4	–	37.7	1.43 (sept, 6.8)
12	30.9	1.87 (s)	32.0	1.88 (s)	18.4	0.76 (d, 6.8)
13	29.8	1.79 (s)	30.7	1.85 (s)	17.2	0.92 (d, 6.8)
14	23.7	1.18 (s)	22.4	1.11 (s)	19.3	1.15 (s)
15	11.6	2.00 (s)	11.2	2.02 (s)	30.2	2.21 (s)

^m^multiplet or overlapped

^a^measured in C_5_D_5_N

^b^measured in CD_3_OD

*The ^13^C NMR data of known compound **1** is reported here for the first time

**Fig. 4:**
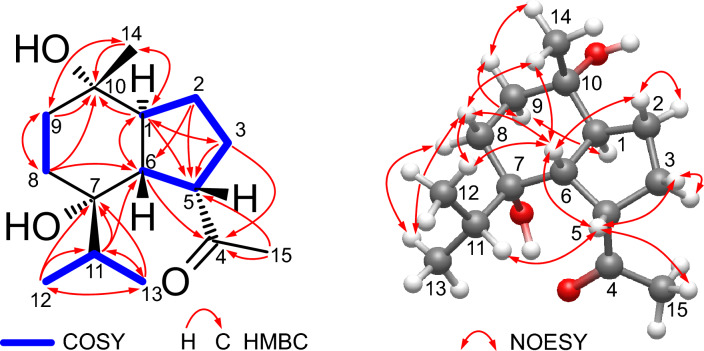
COSY, HMBC and NOESY correlations of **3**

**Fig. 5:**
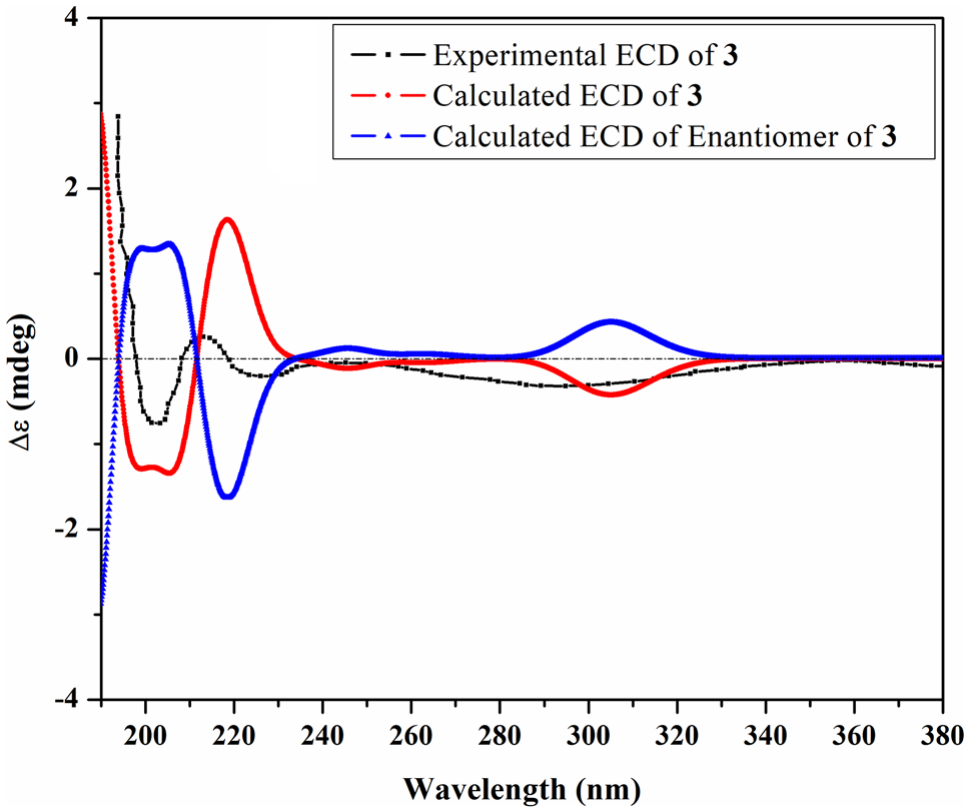
Experimental and TDDFT-simulated ECD spectra of **3**

Compound **3** [*α*]_D_^23^ –3.64 (*c* = 3.3, MeOH) exhibited a molecular formula of C_15_H_26_O_3_ determined by positive ion-mode HR-ESI–MS that showed a sodiated molecular ion peak at *m/z*: 277.1773 [M + Na]^+^ (Calcd for C_15_H_26_O_3_Na^+^: 277.1774). The IR spectrum of compound **3** displayed the existence of hydroxy (3419 cm^−1^) and ketone (1705 cm^−1^) functionalities. The index of hydrogen deficiency suggested a two-ring system. The ^1^H-NMR spectrum of **3** (Table 1) showed the presence of a triplet of doublets signal at *δ*_H_ 2.08 (td, *J* = 12.5, 5.4 Hz, H-1) and a doublet of doublets proton signal at *δ*_H_ 2.15 (*J* = 12.5, 8.8 Hz, H-6). These large coupling constants indicated a *trans*-diaxial linkage of the rings. In addition, two doublet methyls at *δ*_H_ 0.76 (d, *J* = 6.8 Hz, H-12) and *δ*_H_ 0.92 (d, *J* = 6.8 Hz, H-13), and a septet signal at *δ*_H_ 1.43 (sept, *J* = 6.8 Hz, H-11) were assignable for an isopropyl function. Two singlet methyl signals at *δ*_H_ 1.15 and *δ*_H_ 2.21 were assigned for H-14 and H-15, respectively, based on the difference in chemical shift values. The ^13^C-NMR and DEPT spectra (Table 1) showed the presence of 15 carbon signals, indicating the sesquiterpenoid nature of compound **3**. Four methyl carbon signals at *δ*c 18.4, 17.2, 19.3, and 30.2 were assignable for C-12, 13, 14, and 15, respectively. Besides, four methylene carbon signals at *δ*c 26.6, 29.0, 30.4 and 38.1 were assignable for C-2, 8, 3, and 9, respectively. In addition, the ^13^C-NMR revealed the presence of four additional methine carbon signals resonated at *δ*_C_ 51.8 (C-1), 50.7 (C-5), 50.4 (C-6) and 37.7 (C-11), two hydroxylated quaternary carbon signals at *δ*_C_ 75.7 (C-7), 73.7 (C-10) and one ketonic carbonyl carbon at *δ*_C_ 215.1 corresponding to C-4. Full inspection of the compound was carried out through 2D analyses, including ^1^H-^1^H COSY, HSQC and HMBC analyses as shown in Fig. 4. These data were closely related to oplopanane sesquiterpenes [20], except the downfield shift of C-7 to *δ*_C_ 75.6 confirmed the hydroxylation of C-7. The NOESY correlations (Fig. 4) of H-6 with H-5, H-12, and H_3_-14 showed the same orientation of them. The absolute configuration was determined to be 1*S*, 5*S*, 6*R*, 7*S* and 10*S* by ECD calculation, as shown in Fig. 5. Consequently, from the above-mentioned data, the structure of compound **3** was concluded as a previously undescribed compound, namely 5-*epi*-7α-hydroxy-( +)-oplopanone.

### Cytotoxic activity

The growth-inhibitory effect of compounds **1**–**11** of *L. arboreum* on the lung adenocarcinoma (A549), breast cancer (MCF-7) and hepatocellular carcinoma (HepG2) was examined. Compound **9** exhibited potent cytotoxic activity against A549 and MCF-7 cell lines (IC_50_ = 16.5 ± 1.3 and 24.7 ± 2.2 μg/ml, respectively) as compared with the standard antitumor agent etoposide (IC_50_ 28.4 ± 4.5 and 22.2 ± 4.2 μg/mL, respectively).

Compounds **4** and **8** showed potent cytotoxicity against the A549 cell line (IC_50_ = 17.0 ± 2.5, and 13.5 ± 2.1 μg/ml, respectively) and moderate cytotoxic activities toward the MCF-7 cell line (IC_50_ = 35.6 ± 1.9 and 33.1 ± 1.0 μg/mL, respectively), and the HepG2 cell line (IC_50_ = 34.7 ± 1.5, and 36.7 ± 1.6 μg/mL, respectively). The remaining compounds showed very weak or no activity toward the tested cell lines (Table 2).

**Table 2:** Cytotoxic and anti-leishmanial activities of **1**–**11** from *L. arboretum*

No	IC_50_ (μg/ml)			
	A549	MCF-7	HepG2	*L. major*
1	> 100	> 100	> 100	> 100
2	> 100	> 100	> 100	> 100
3	> 100	> 100	> 100	> 100
4	17.0 ± 2.5	35.6 ± 1.9	34.7 ± 1.5	> 100
5	> 100	> 100	> 100	70.2 ± 1.5
6	83.8 ± 3.45	> 100	> 100	> 100
7	> 100	> 100	> 100	> 100
8	13.5 ± 2.1	33.1 ± 1.0	36.7 ± 1.6	67.4 ± 2.4
9	16.5 ± 1.3	24.7 ± 2.1	59.6 ± 1.9	77.5 ± 1.3
10	69.7 ± 1.3	74.9 ± 1.11	70.9 ± 2.1	> 100
11	84.0 ± 2.5	91.8 ± 1.2	87.7 ± 1.8	> 100
Etoposide	28.4 ± 4.5	22.2 ± 4.2	20.2 ± 0	–
Miltefosine	–	–	–	7.7 ± 2.1

*–* not determined, *n* = 3

### Anti-leishmanial activity

The growth-inhibitory effects of the isolated compounds on the *L. major* promastigotes were assessed. Compounds **5**, **8**, and **9** showed weak anti-leishmanial activity with IC_50_ values of 70.2 ± 1.5, 67.4 ± 2.4, and 77.5 ± 1.3 μg/mL, respectively, compared to the standard anti-leishmanial agent miltefosine (IC_50_ 7.7 ± 2.1 μg/mL), although the other compounds showed no anti-leishmanial activity (Table 2).

## Experimental

### General experimental procedures

^1^H and ^13^C-NMR spectra were measured on a JEOL spectrometer at 600 and 150 MHz, respectively. HR-ESI–MS data were noted on a Thermo Fisher Scientific LTQ Orbitrap XL spectrometry. Silica gel for column chromatography (70–230) was used for fine separation (E. Merck, Darmstadt, Germany). Silica gel 60 pre-coated plates F254 (E. Merck, Germany) were used for TLC detection. Reversed-phase (RP-C_18_) silica gel purchased from Nacalai Tesque, Kyoto, Japan was utilized for reversed-phase column chromatography separation. Inertsil ODS-3 column (GL Science, Tokyo, Japan) was used for HPLC analyses using a refractive index detector (RID-6A, Shimadzu, Kyoto, Japan). The stable 3D molecular models were obtained using the MMFF94s force field.

The cancer-cell lines (lung adenocarcinoma (A549), breast cancer (MCF-7), and hepatocellular carcinoma (HepG2)) were obtained from the National Institute of Biomedical Innovation's Japanese Collection of Research Bioresources (JCRB) Cell Bank. The Institute of Tropical Medicine at Nagasaki University in Japan provides *Leishmania major.* Dimethyl sulfoxide, Dulbecco’s modified Eagleʹs medium, fetal bovine serum, medium 199, miltefosine, etoposide, 3-(4,5-dimethythiazol-2-yl)-2,5-diphenyltetrazolium bromide (MTT), and kanamycin were obtained from Nacalai Tesque, Kyoto, Japan. Becton Dickinson provided the 96-well plates (Franklin Lakes, NJ, USA).

### Soft coral material

The *Litophyton arboreum* soft coral was collected at a depth of 10–15 m using a diving technique (SCUBA) in March 2018 in front of the National Institute of Oceanography and Fisheries at Hurghada province on the Egyptian Red Sea Coast. The sample was collected and identified by Abdallah Alian Department of Zoology, Faculty of Science, Al-Azhar University, Assiut-Branch, Assiut, Egypt. The sample was stored in a freezer until the time of extraction. A voucher specimen was deposited with the symbol of LA.5 at the Department of Zoology, Faculty of Science, Al-Azhar University, Assiut-Branch, Assiut, Egypt.

### Extraction and isolation

*L. arboreum* ( ~ 1.8 kg wet wt.) was cut into small pieces and macerated in methanol until it was exhausted. The methanolic extract has been concentrated using reduced pressure to get a dried residue (38 g). The total methanolic extract was fractionated using vacuum liquid-column chromatography (CC) filled with silica gel. The elution was carried out with the solvents [*n*-hexane (3L), *n*-hexane-chloroform (1:1) (3L), chloroform (3L), EtOAc (3L), and MeOH (3L)], successively, produced *n*-hexane (1.0 g), *n*-hexane-chloroform (1:1) (9.0 g), chloroform (2.5 g), EtOAc (5.5 g), and MeOH (18.0 g) fractions.

The *n*-hexane-chloroform (1:1) fraction was subjected to silica gel CC and eluted with EtOAc in *n*-hexane (0–100% of EtOAc gradients) and further with MeOH in EtOAc (0–100% of MeOH gradients) and yielded 22 sub-fractions (F1-F22).

The sub-fraction F3 (3.5 g), eluted with *n*-hexane–EtOAc (80:20), was chromatographed over reversed-phase CC and eluted with MeOH-H_2_O (0–100% of MeOH gradients) to afford ten sub-fractions (F3-1⁓F3-10). The subfraction F3-5 (415 mg) eluted with 50% MeOH, was finally purified on a reversed-phase HPLC with MeOH–H_2_O, 50:50, and afforded compounds **1** (7.0 mg) and **2** (4.0 mg). The subfraction F3-6 (400 mg) eluted with 50% MeOH, was finally purified on a reversed-phase HPLC with MeOH–H_2_O, 60:40, and afforded compound **4** (40.0 mg).

The sub-fraction F5 (2.2 g), eluted with *n*-hexane–EtOAc (60:40), was chromatographed over reversed-phase CC and eluted with MeOH-H_2_O (0–100% of MeOH gradients) to afford ten sub-fractions (F5-1⁓F5-10). The subfraction F5-4 (200 mg) eluted with 40% MeOH, was finally purified on a reversed-phase HPLC with MeOH–H_2_O, 40:60, and afforded compound **5** (10.5 mg). The subfraction F5-5 (240 mg) eluted with 50% MeOH, was finally purified on a reversed-phase HPLC with MeOH–H_2_O, 50:50, and afforded compounds **6** (7.7 mg) and **7** (24.0 mg). The subfraction F5-9 (879 mg) eluted with 90% MeOH, was finally purified on a reversed-phase HPLC with MeOH–H_2_O, 90:10, and afforded compounds **8** (17.6 mg), **9** (5.6 mg), **10** (11.5 mg) and **11** (20.0 mg).

The sub-fraction F9 (710.8 g), eluted with *n*-hexane–EtOAc (20:80), was chromatographed over reversed-phase CC and eluted with MeOH-H_2_O (0–100% of MeOH gradients) to afford ten sub-fractions (F9-1⁓F9-10). The subfraction F9-5 (150 mg) eluted with 50% MeOH, was finally purified on a reversed-phase HPLC with MeOH–H_2_O, 50:50, and afforded compound **3** (3.5 mg).

### 8β,11-Dihydroxy-β-cyperon (2)

Colorless oil; [α]_D_^23^ + 96.15 (*c* 2.6, MeOH); UV λ_max_ nm (log ɛ) (MeOH): 296 (4.04); IR (film) *v*_max_ 3393, 2965, 1658, 1541, 1374, 1132, 1095, 1071, 1032 cm^−1^; ^1^H NMR (C_5_D_5_N, 600 MHz) and ^13^C NMR (C_5_D_5_N, 150 MHz): see Table 1; HR-ESI–MS (positive ion-mode) *m/z*: 273.1461 [M + Na]^+^ (Calcd 273.1461 for C_15_H_22_O_3_Na).

### 5-epi-7α-Hydroxy-( +)-oplopanone (3)

Colorless viscous oil; [α]_D_^23^ -3.64 (*c* 3.3, MeOH); IR (film) *v*_max_ 3419, 2936, 1705, 1374, 1132, 1033, 501 cm^−1^; ^1^H NMR (CD_3_OD, 600 MHz) and ^13^C NMR (CD_3_OD, 150 MHz): see Table 1; HR-ESI–MS (positive ion-mode) *m/z*: 277.1773 [M + Na]^+^ (Calcd 277.1774 for C_15_H_26_O_3_Na).

### Evaluation of cytotoxicity

Cytotoxic activity was determined against different cell lines, A549, MCF-7 and HepG2, using the colorimetric cell viability MTT method described previously [21, 22].

### Evaluation of antileishmanial assay

The antileishmanial action was assessed using MTT colorimetric cell viability assay method described previously [23, 24].

### Density functional theory calculations

Using Omega2 software (OMEGA, 2.5.1.4; OpenEye Scientific Software: Santa Fe, NM, USA, 2013), conformational analysis was executed to unveil the possible conformers within an energy window of 10 kcal/mol for compounds 2 and 3. All generated conformers were optimized employing the B3LYP/6-31G* level of theory with the aid of Gaussian09 software [25]. Relying on the optimized structures, frequency computations were performed to reveal the nature of the local minimum of the inspected structures and estimate the corresponding Gibbs free energies. Employing a polarizable continuum model (PCM), time-dependent density functional theory (TDDFT) calculations were established at the B3LYP/6-31G* level of theory utilizing methanol as a solvent to compute the first fifty excitation states. Utilizing Gaussian band shapes with sigma = 0.20–30 eV, electronic circular dichroism (ECD) spectra were finally generated with the help of SpecDis 1.71 software (SpecDis 2017) [26, 27]. The extracted ECD spectra were subjected to Boltzmann average.

## Conclusions

Chemical investigation of Red Sea soft coral *Litophyton arboreum* resulted in the isolation of eleven compounds, including two previously undescribed sesquiterpenes*. In* addition, the isolated compounds exhibited potent cytotoxic and anti-leishmanial activities. Therefore, these compounds were considered promising candidates for anticancer and anti-leishmanial reagents, although *f*urther chemical and biologic investigations are needed.

## Supplementary Information

Below is the link to the electronic supplementary material.

Supplementary file1 (PDF 2432 KB)
