# Supplementary material for: New sesquiterpenes from the soft coral Litophyton arboreum
Source: J Nat Med. 2024 Oct 22;79(1):162–9. doi: 10.1007/s11418-024-01843-w (PMC11735488; doi:10.1007/s11418-024-01843-w)
Supplement: Supplementary file 1 — Supplementary file1 (PDF 2432 KB) [file 11418_2024_1843_MOESM1_ESM.pdf]

Supplementary Material

New sesquiterpenes from the soft coral *Litophyton arboreum*

Amany Hamouda Mahmoud[1,2], Sabry A. H. Zidan[3], Mamdouh Nabil Samy[2], Abdallah Alian[4], Mahmoud A. A. Ibrahim[5,6], Mostafa Ahmed Fouad[2], Mohamed Salah Kamel[2,*], Katsuyoshi Matsunami[1,*]

[1]Department of Pharmacognosy, Graduate School of Biomedical and Health Sciences, Hiroshima University, Hiroshima, Japan

[2]Department of Pharmacognosy, Faculty of Pharmacy, Minia University, 61519 Minia, Egypt

[3]Department of Pharmacognosy, Faculty of Pharmacy, Al-Azhar University, Assiut-Branch, Assiut 71524, Egypt

[4]Department of Zoology, Faculty of Science, Al-Azhar University, Assiut-Branch, Assiut 71524, Egypt

[5]Chemistry Department, Faculty of Science, Minia University, 61519 Minia, Egypt.

[6]School of Health Sciences, University of KwaZulu-Natal, Westville, Durban 4000, South Africa

*Correspondences: matunami@hiroshima-u.ac.jp (KM), mskamel@yahoo.com (MSK)

Abstract

Two new sesquiterpenes; 8α,11-dihydroxy-β-cyperon (**2**), and 5-*epi*-7α-hydroxy-(+)-oplopanone (**3**), were isolated from the soft coral *Litophyton arboreum,* together with nine known ones, including five sesquiterpenes; 11-hydroxy-8-oxo-β-cyperon (**1**), alismoxide (**4**), 5β,8β-epidioxy-11-hydroxy-6-eudesmene (**5**), chabrolidione B (**6**), 7-oxo-tri-nor-eudesm-5-en-4β-ol (**7**), two sterols; 7β-acetoxy-24-methyl-cholesta-5,24(28)-diene-3β,19-diol (**8**), nebrosteroid M (**9**), and two glycerol derivatives; chimyl alcohol (**10**) and

batyl alcohol (**11**). The structures of the isolated compounds were characterized using spectroscopic techniques, predominately HR-ESI-MS, 1D, 2D-NMR and ECD analyses. Compounds **1**-**11** were evaluated for their cytotoxic activity against three human cancer cell lines (A549, MCF-7 and HepG2), and anti-leishmanial potential against the causal parasite, *Leishmania major.* Compounds **4**, **8**, and **9** exhibited potent cytotoxic activity against the A549 cell line (IC_{50} = 17.0±2.5, 13.5±2.1, and 16.5±1.3 μg/ml, respectively) as compared with the standard antitumor agent etoposide (IC_{50} 28.4±4.5). Additionally, compound **9** exhibited remarkable cytotoxic activity against MCF-7 cell line (IC_{50} = 24.7±2.1 μg/ml: 22.2±4.2 μg/mL for etoposide).

Keywords: *Litophyton arboreum* · Nephtheidae · soft corals · sesquiterpenes · cytotoxicity · anti-leishmania

* To whom all correspondence should be addressed

· Katsuyoshi Matsunami

Graduate School of Biomedical and Health Sciences, Hiroshima University, 1-2-3 Kasumi, Minami-ku, Hiroshima 734-8553, Japan

TEL&FAX: +81-82-257-5335

e-mail: matunami@hiroshima-u.ac.jp.

· Mohamed Salah Kamel

Department of Pharmacognosy, Faculty of Pharmacy, Minia University, 61519 Minia, Egypt
e-mail: mskamel@yahoo.com

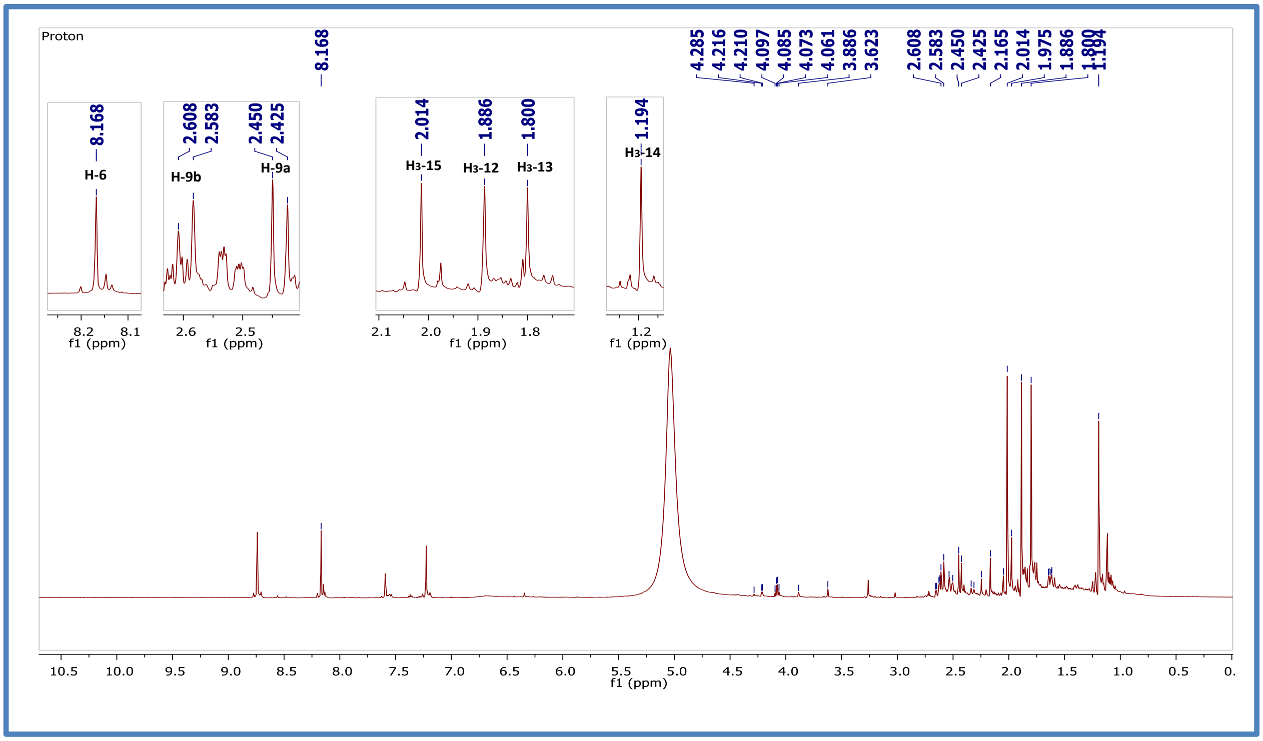

Figure S1: ^{1}H-NMR spectrum of compound 1 (600 MHz, C_5D_5N)

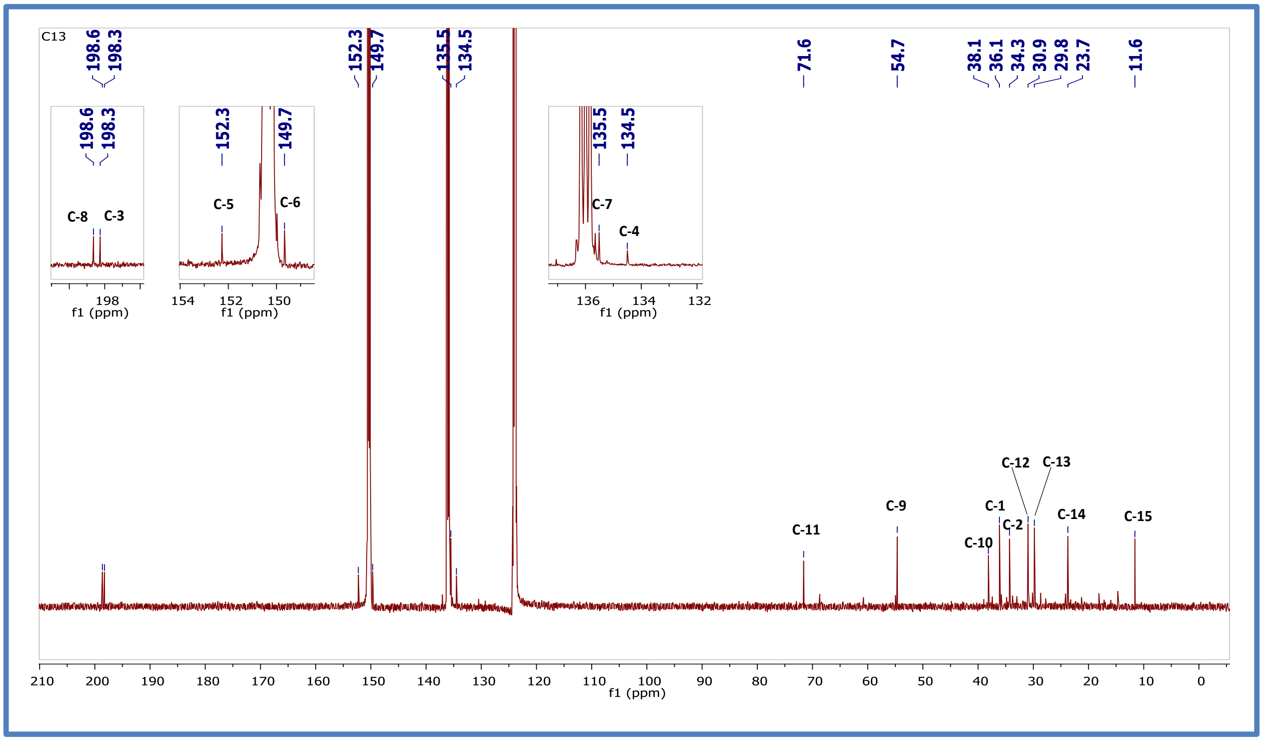

Figure S2: ^{13}C-NMR spectrum of compound 1 (150 MHz, C_5D_5N)

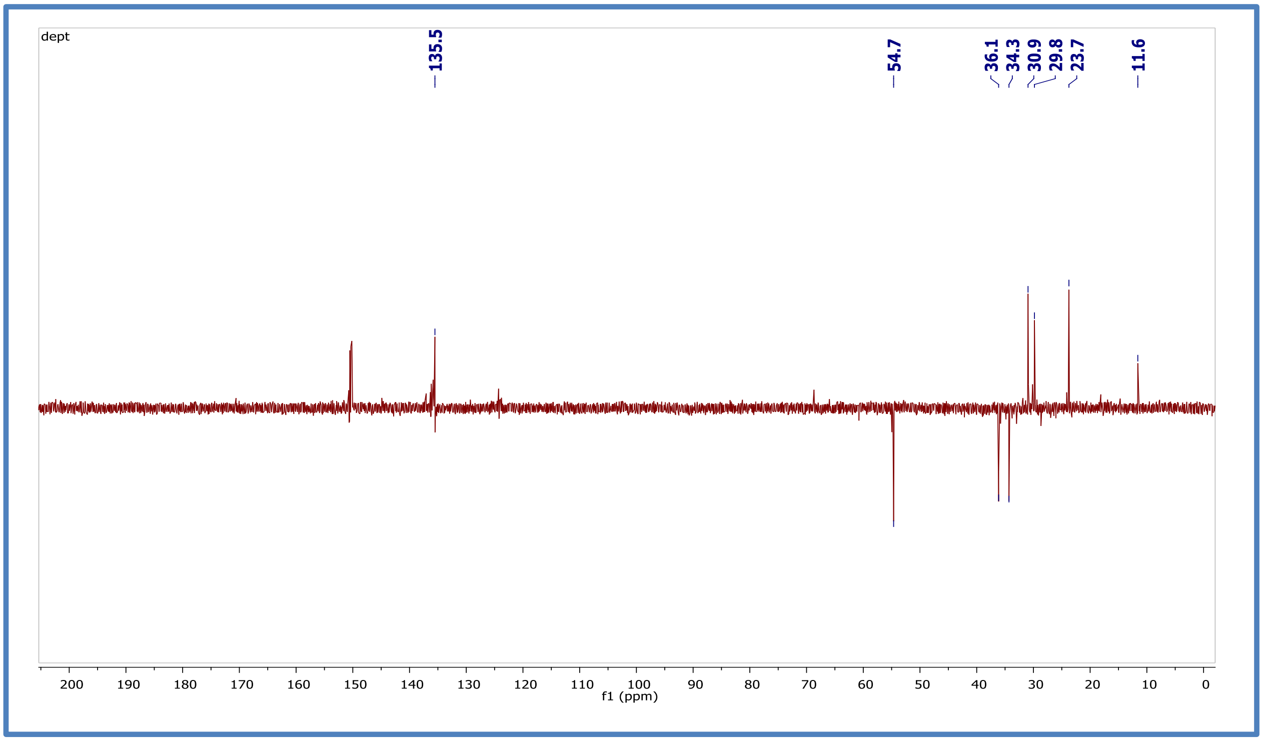

Figure S3: DEPT spectrum of compound 1 (150 MHz, C_5D_5N).

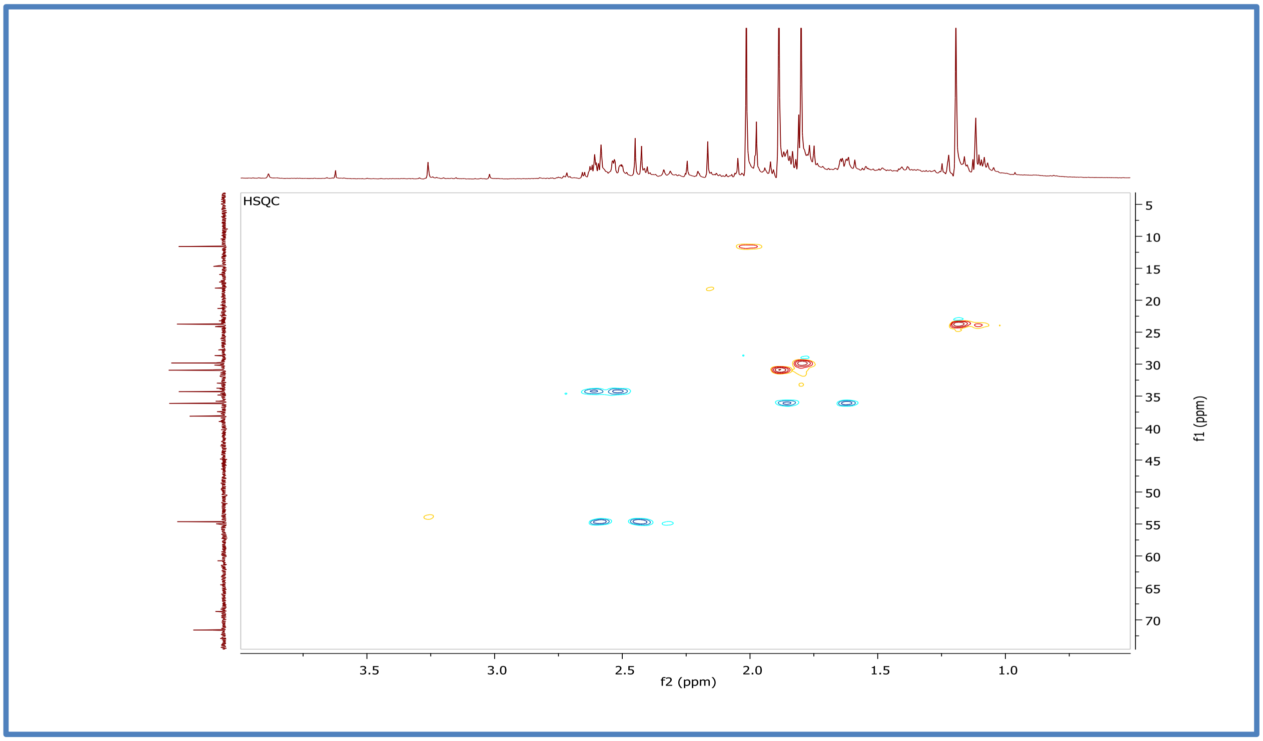

Figure S4: HSQC spectrum of compound 1

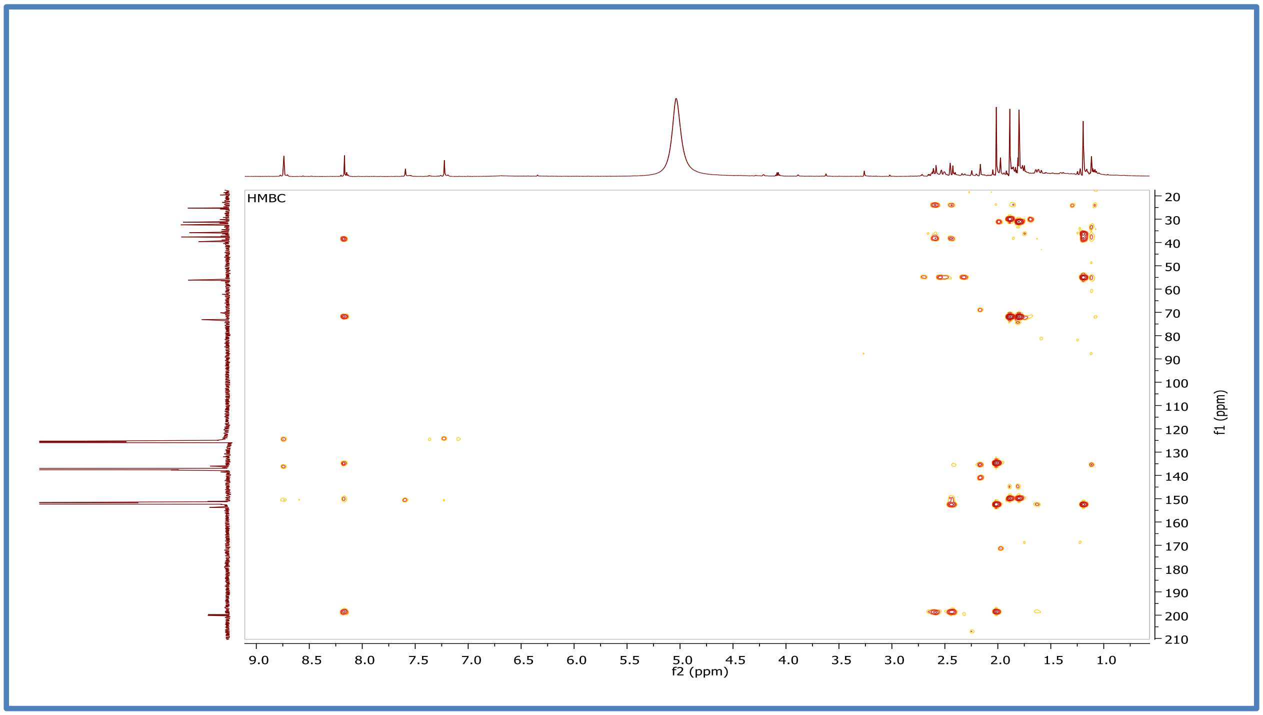

Figure S5: HMBC spectrum of compound 1

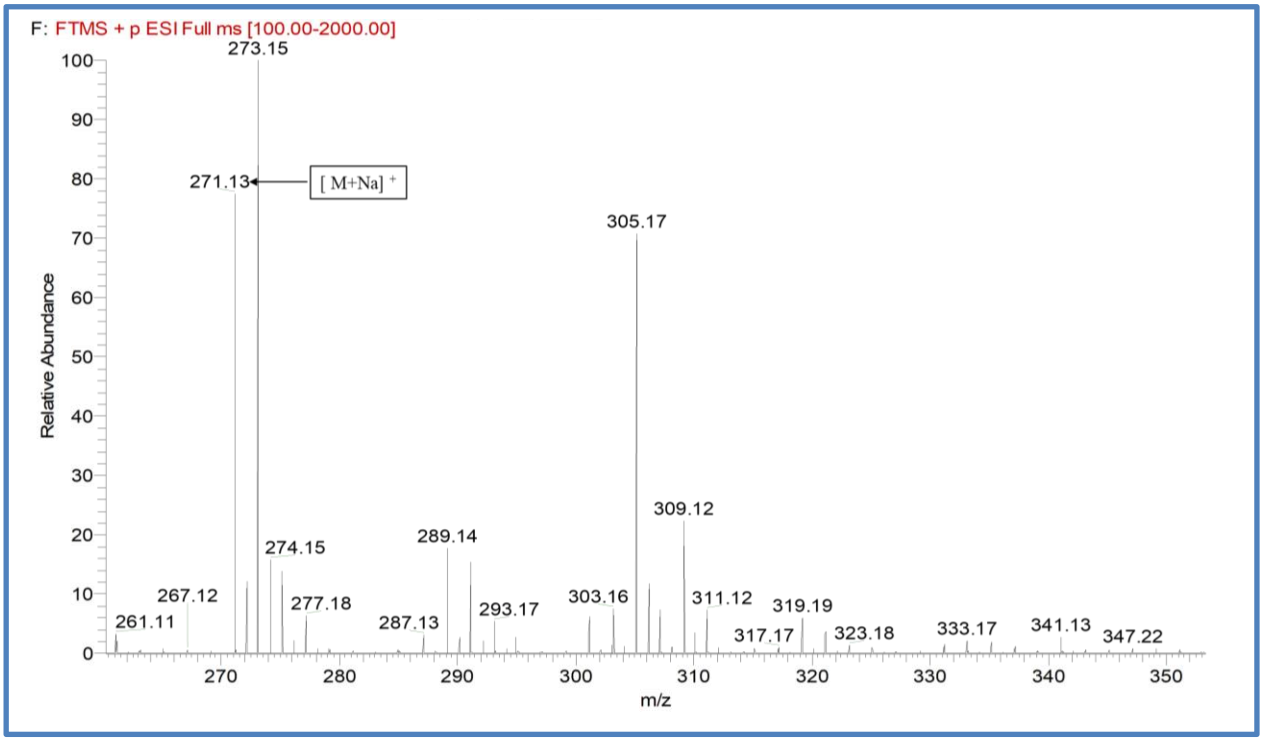

Figure S6: HR-ESI-MS (positive mode) spectrum of compound 1

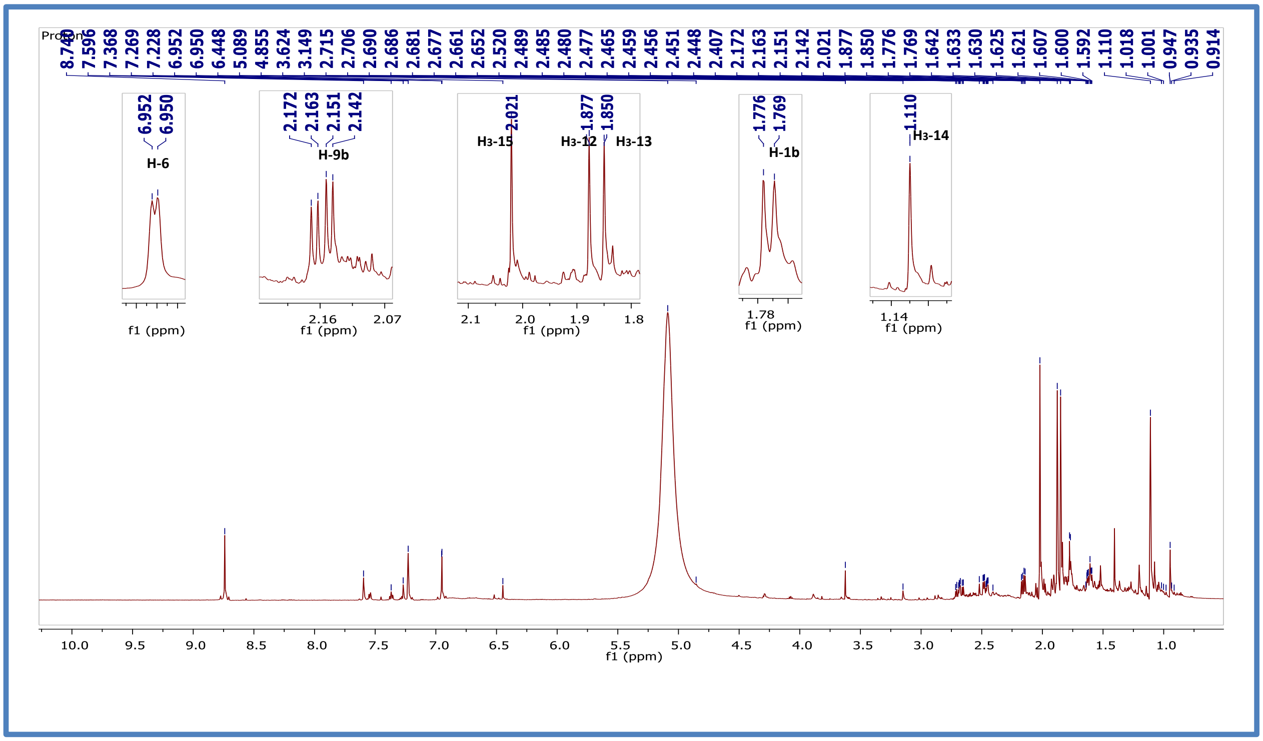

Figure S7: ^{1}H-NMR spectrum of compound 2 (600 MHz, C_5D_5N)

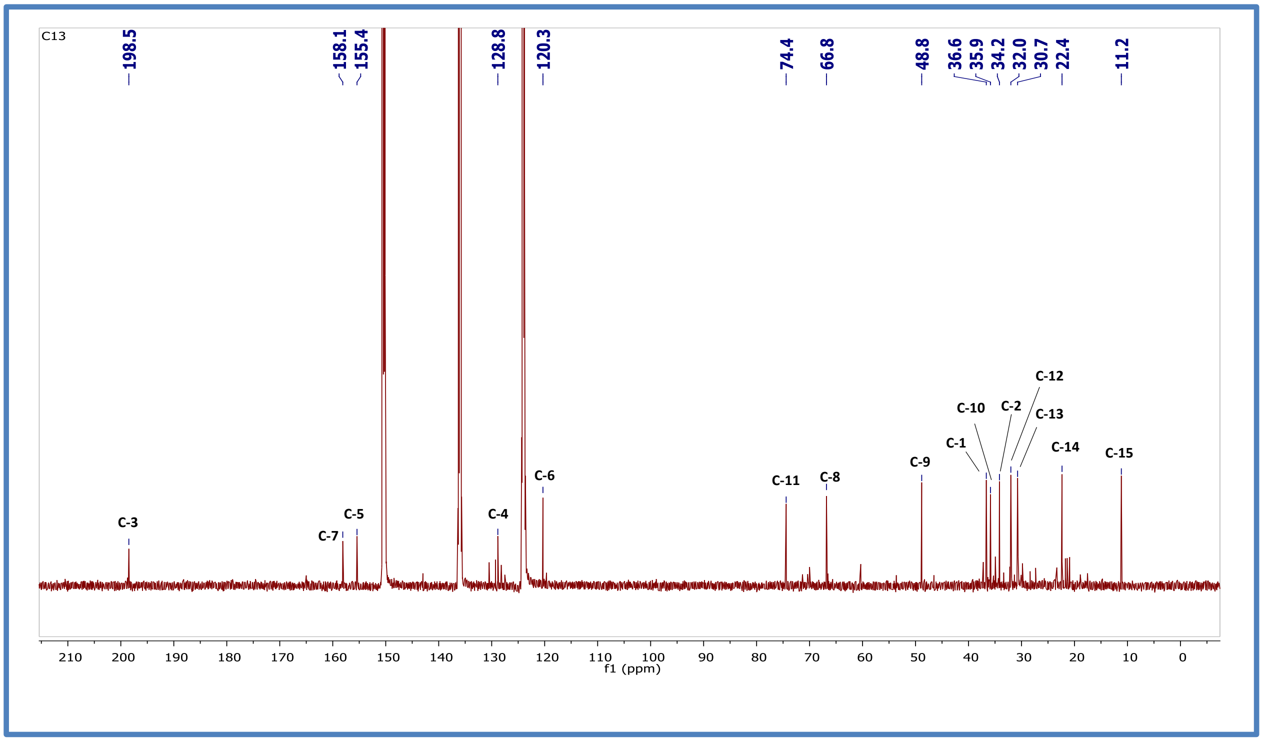

Figure S8: ^{13}C-NMR spectrum of compound 2 (150 MHz, C_5D_5N)

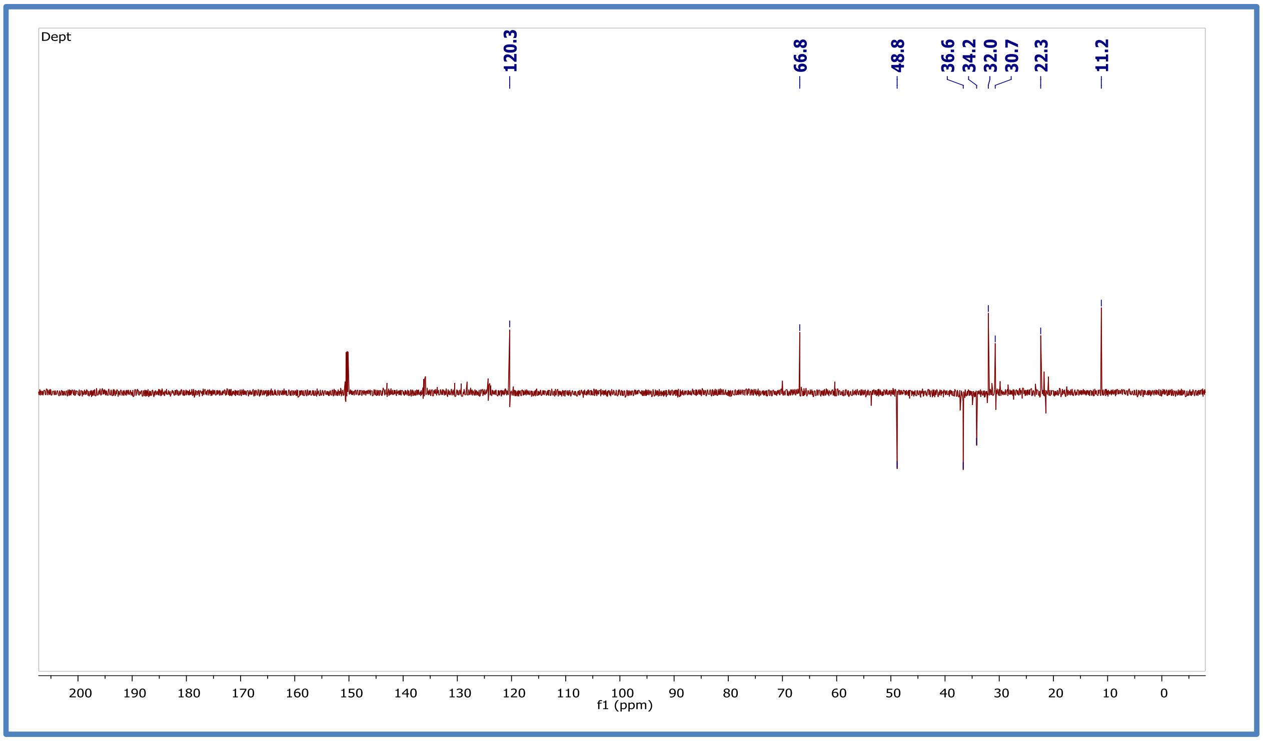

Figure S9: DEPT spectrum of compound 2 (150 MHz, C_5D_5N)

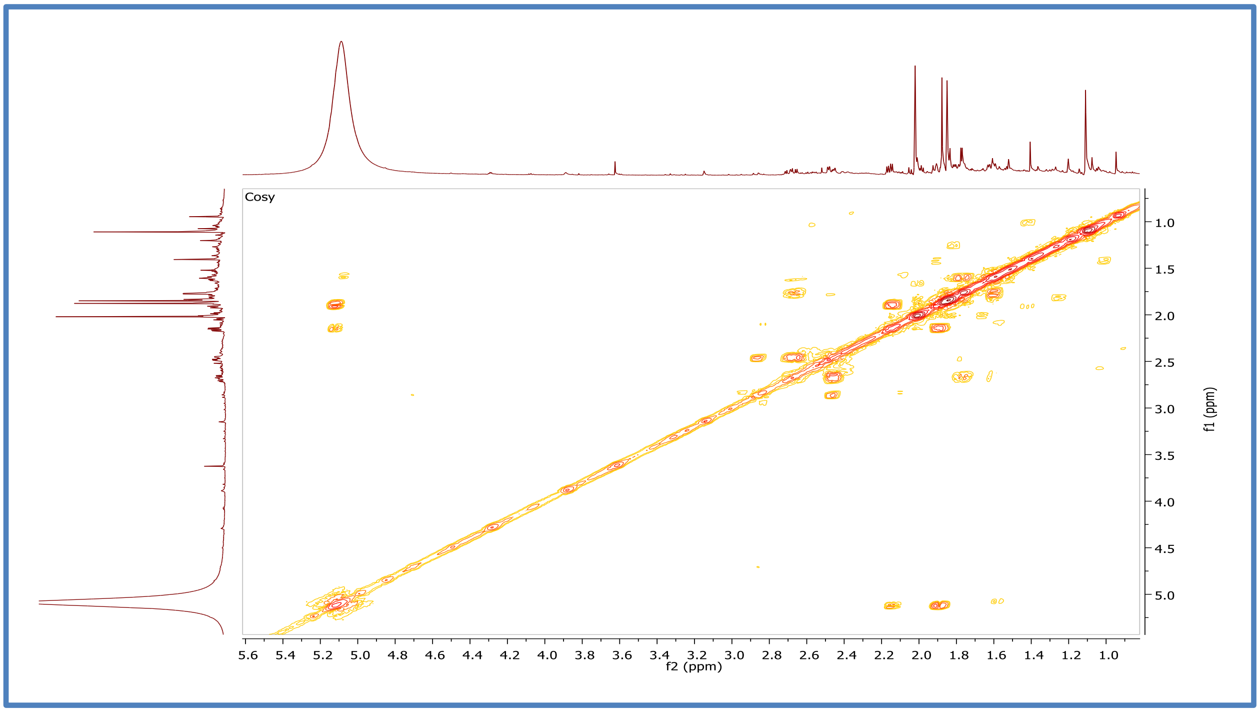

Figure S10: ^1H-^1H COSY spectrum of compound 2

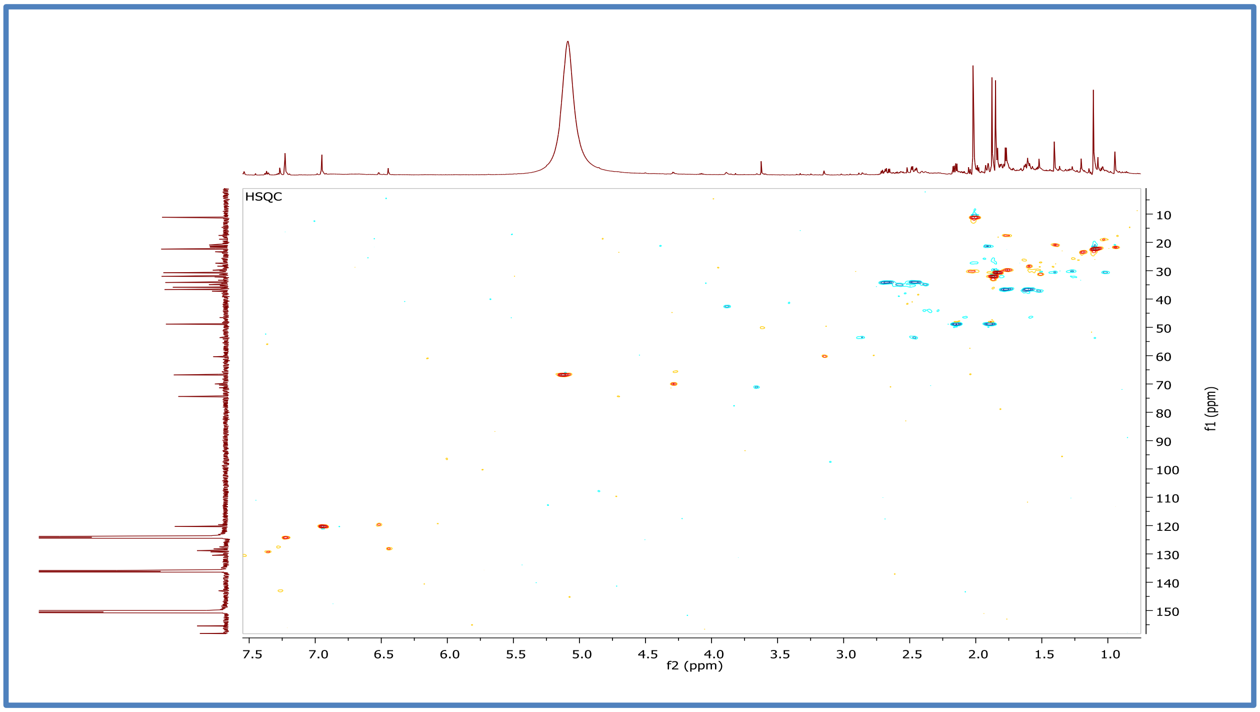

Figure S11: HSQC spectrum of compound 2

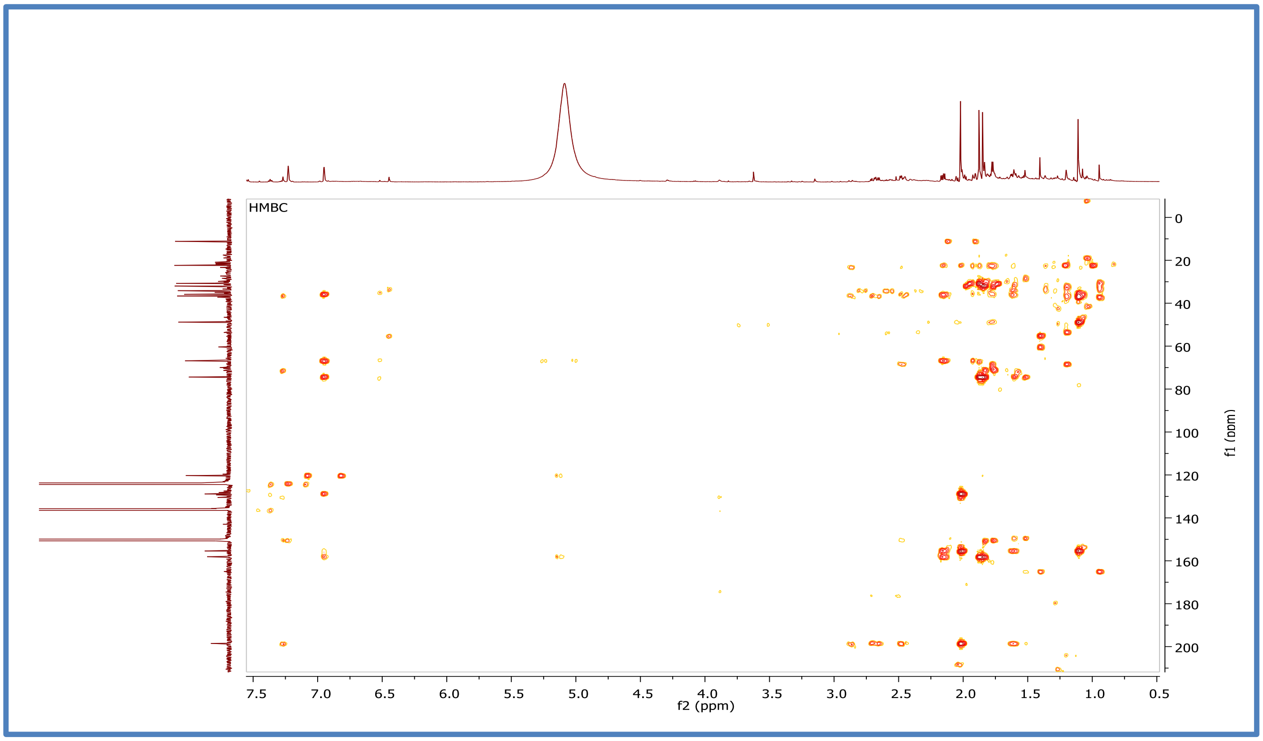

Figure S12: HMBC spectrum of compound 2

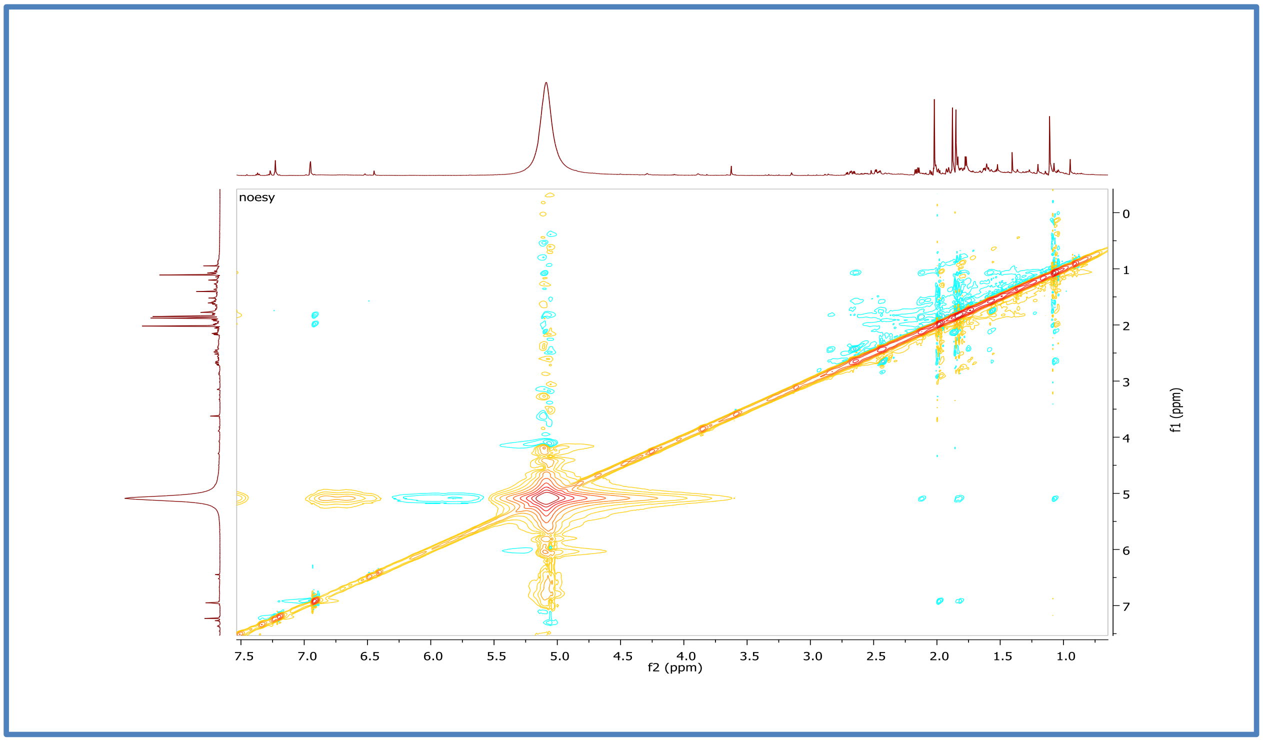

Figure S13: NOESY spectrum of compound 2

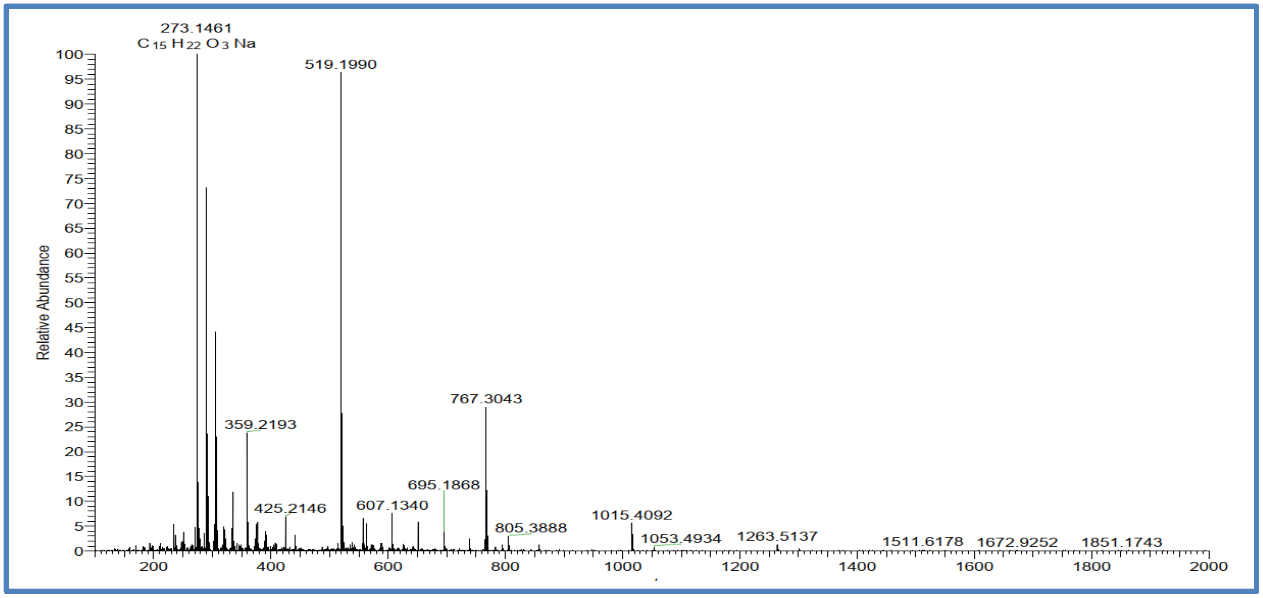

Figure S14: HR-ESI-MS (positive mode) of compound 2

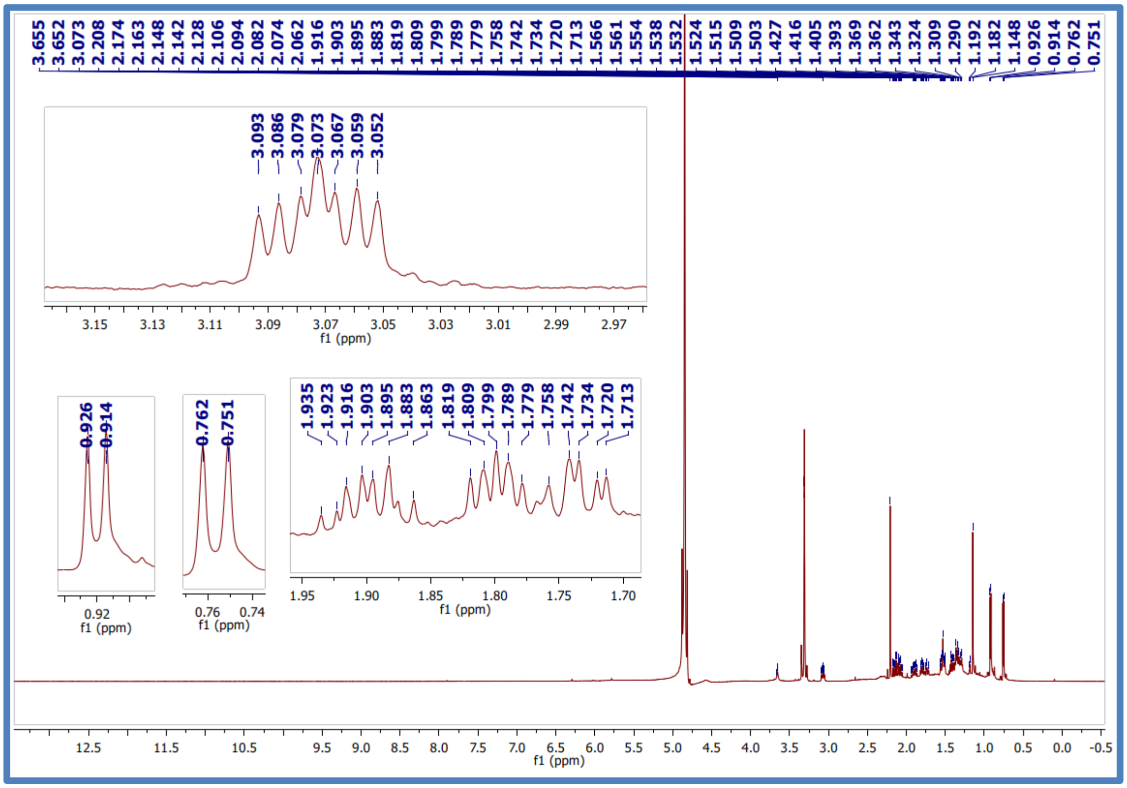

Figure S15: ^{1}H NMR spectrum of compound 3 (150 MHz, CD_3OD)

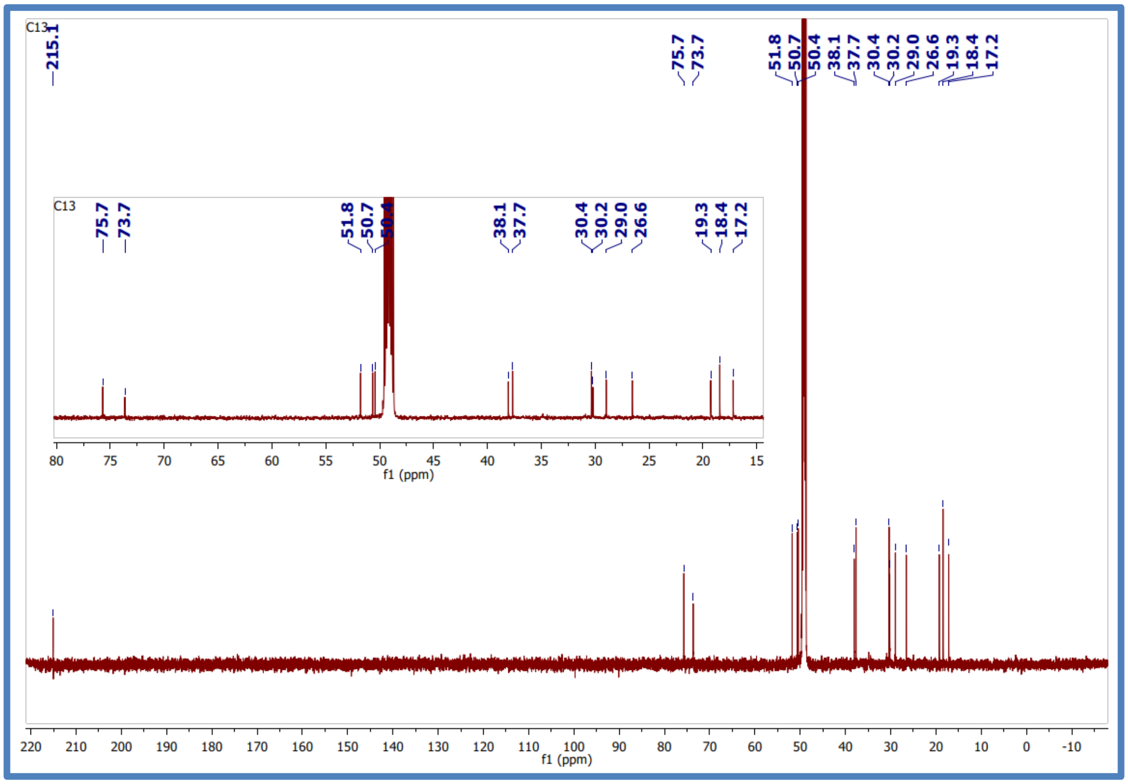

Figure S16: ^{13}C-NMR spectrum of compound 3 (150 MHz, CD_3OD)

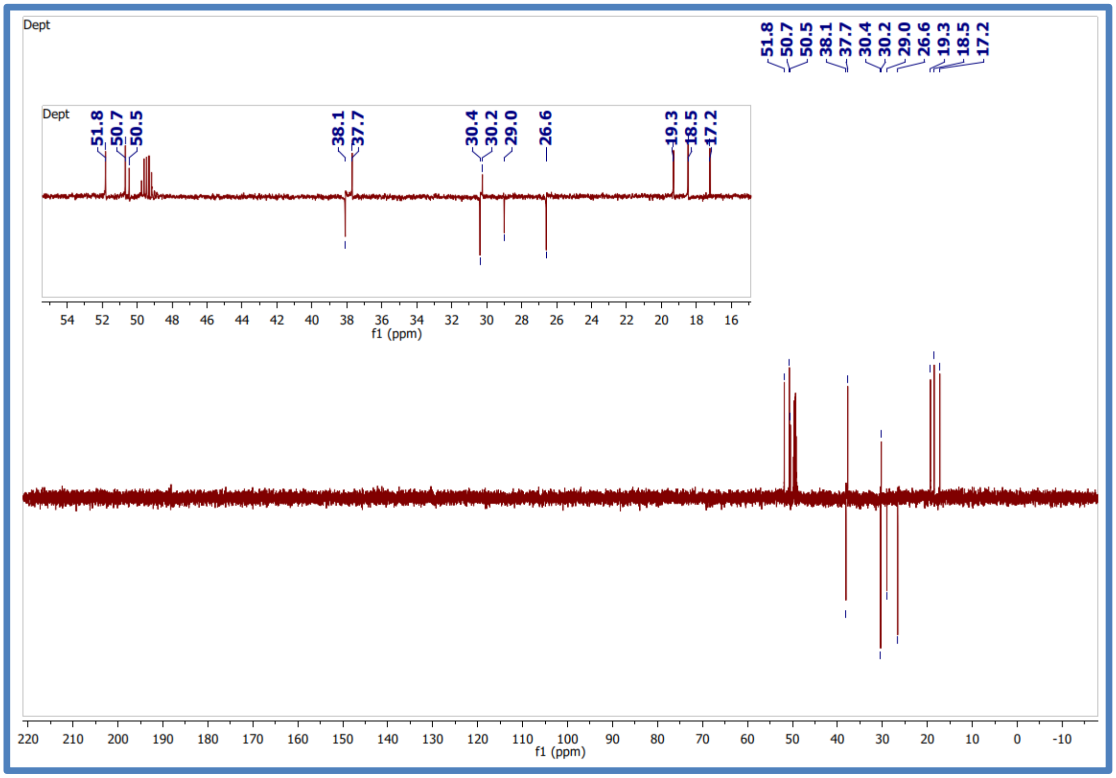

Figure S17: DEPT spectrum of compound 3 (150 MHz, CD_3OD)

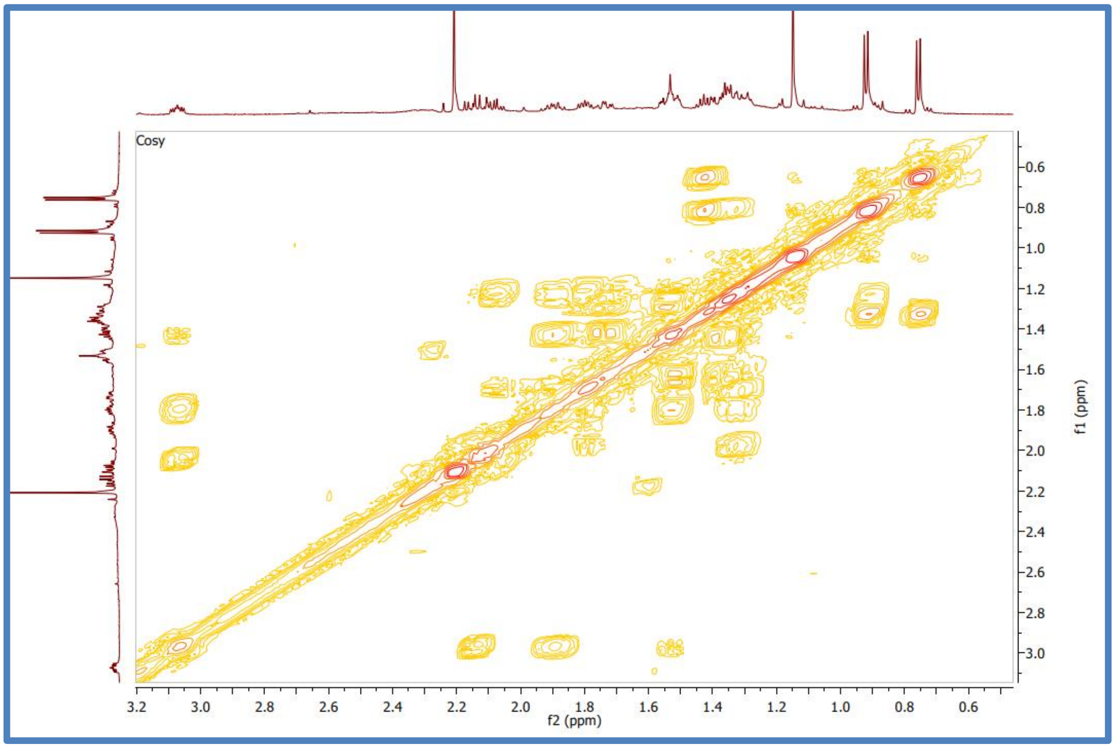

Figure S18: ^{1}H-^{1}H COSY spectrum of compound 3 (150 MHz, CD_3OD)

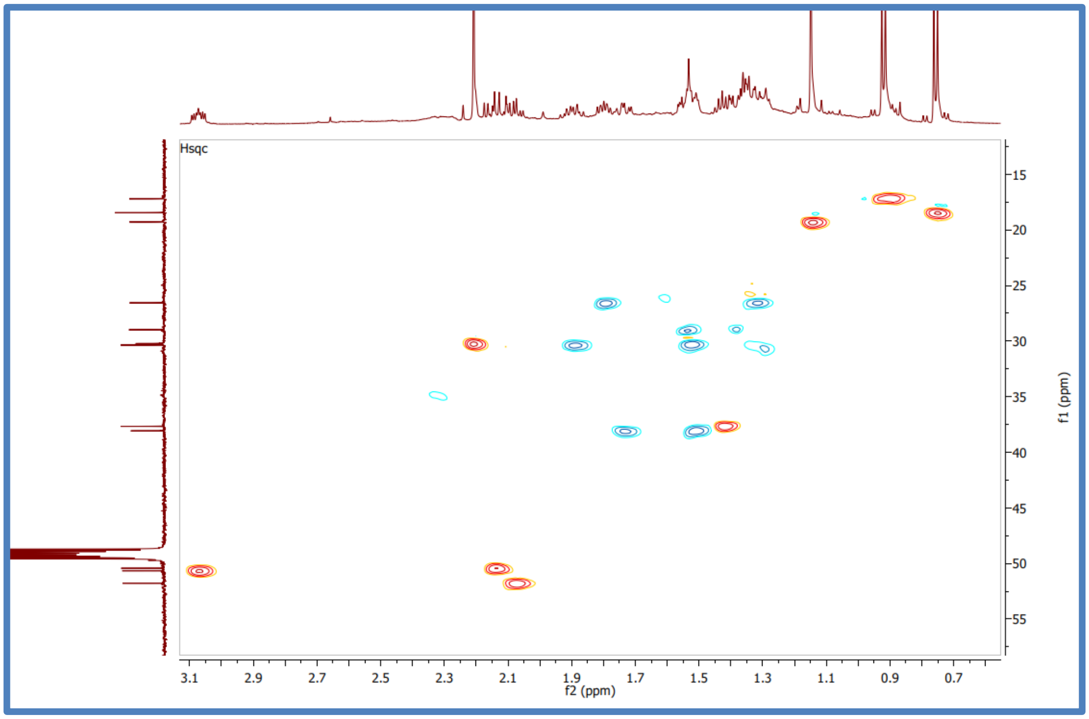

Figure S19: HSQC spectrum of compound 3 (150 MHz, CD_3OD)

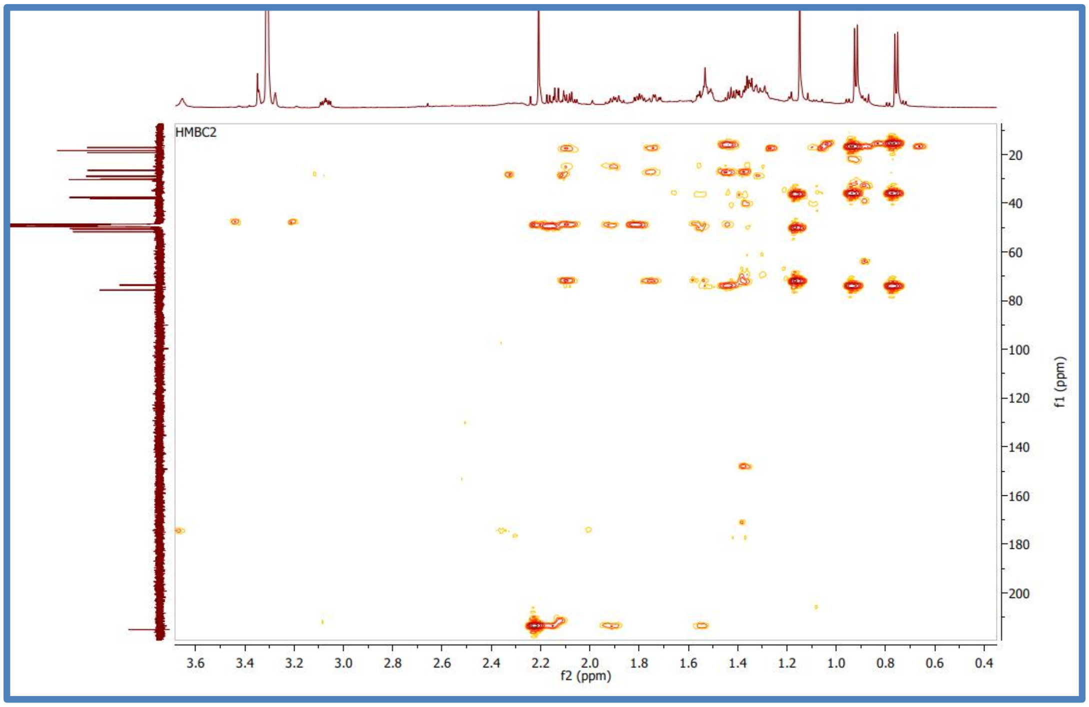

Figure S20: HMBC spectrum of compound 3 (150 MHz, CD_3OD)

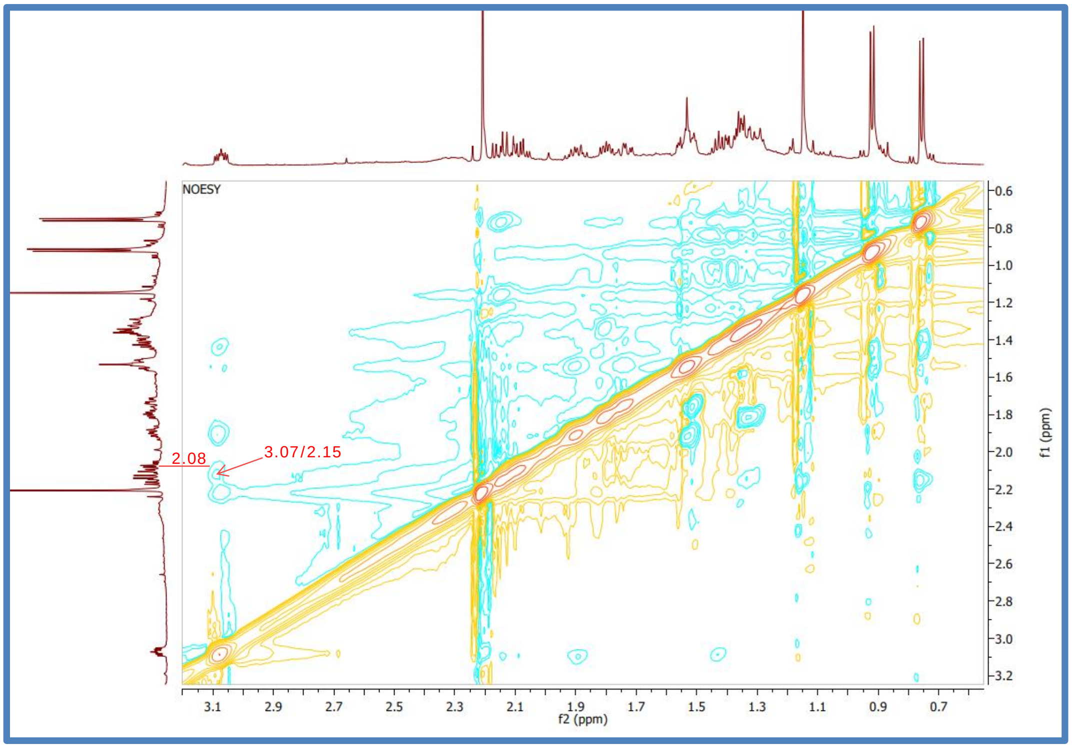

Figure S21: NOESY spectrum of compound 3 (150 MHz, CD_3OD)

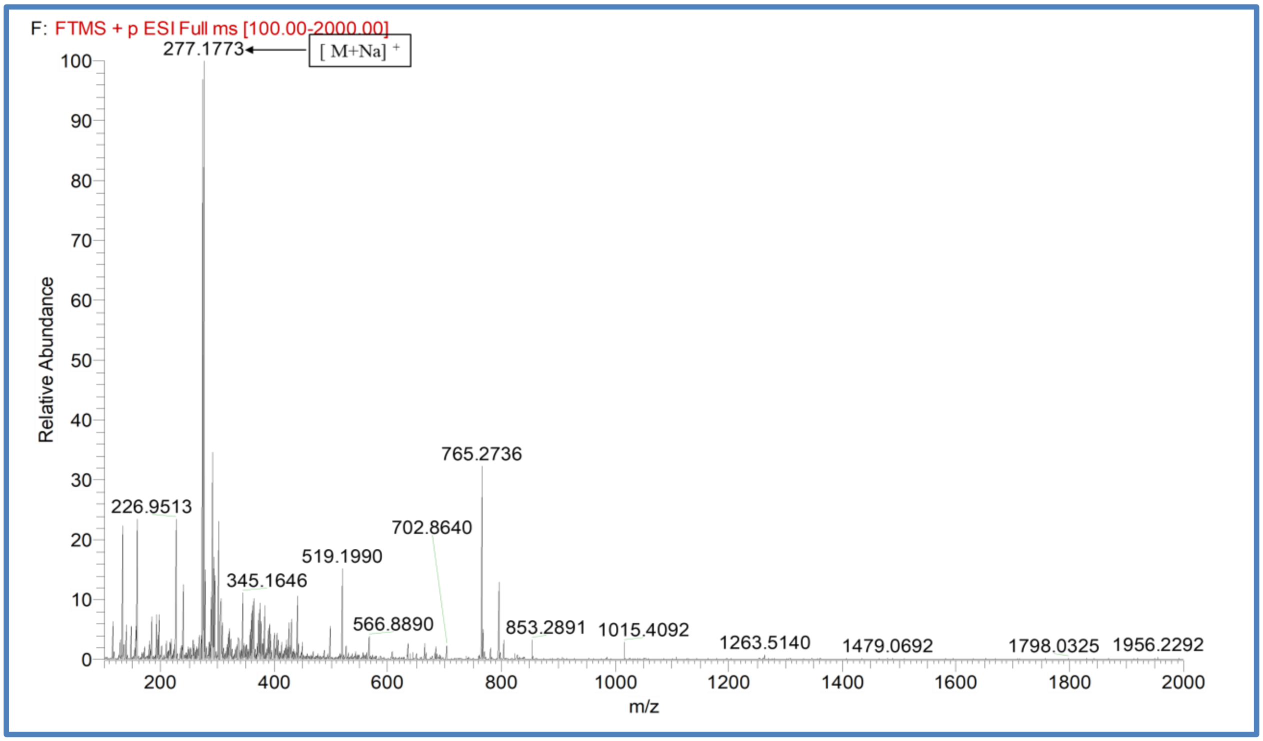

Figure S22: HR-ESI-MS (positive mode) spectrum of compound 3
